# Supervised Machine Learning Based Noninvasive Prediction of Atrial Flutter Mechanism from P-to-P Interval Variability under Imbalanced Dataset Conditions

**DOI:** 10.1155/2023/8162325

**Published:** 2023-03-01

**Authors:** Muhammad Usman Gul, Muhammad Haziq Kamarul Azman, Kushsairy Abdul Kadir, Jawad Ali Shah, Seada Hussen

**Affiliations:** ^1^Universiti Kuala Lumpur, British Malaysian Institute, Kuala Lumpur, Malaysia; ^2^Islamic International University, Islamabad, Pakistan; ^3^School of Electrical and Computer Engineering, Haramaya Institute Technology, Diredawa 138, Ethiopia

## Abstract

Atrial flutter (AFL) is a common arrhythmia with two significant mechanisms, namely, focal (FAFL) and macroreentry (MAFL). Discrimination of the AFL mechanism through noninvasive techniques can improve radiofrequency ablation efficacy. This study aims to differentiate the AFL mechanism using a 12-lead surface electrocardiogram. P-P interval series variability is hypothesized to be different in FAFL and MAFL and may be useful for discrimination. 12-lead ECG signals were collected from 46 patients with known AFL mechanisms. Features for a proposed classifier are extracted through descriptive statistics of the interval series. On the other hand, the class ratio of MAFL and FAFL was 41 : 5, respectively, which was highly imbalanced. To resolve this, different data augmentation techniques (SMOTE, modified-SMOTE, and smoothed-bootstrap) have been applied on the interval series to generate synthetic interval series and minimize imbalance. Modification is introduced in the classic SMOTE technique (modified-SMOTE) to properly produce data samples from the original distribution. The characteristics of modified-SMOTE are found closer to the original dataset than the other two techniques based on the four validation criteria. The performance of the proposed model has been evaluated by three linear classifiers, namely, linear discriminant analysis (LDA), logistic regression (LOG), and support vector machine (SVM). Filter and wrapper methods have been used for selecting relevant features. The best average performance was achieved at 400% augmentation of the FAFL interval series (90.24% sensitivity, 49.50% specificity, and 76.88% accuracy) in the LOG classifier. The variation of consecutive P-wave intervals has been shown as an effective concept that differentiates FAFL from MAFL through the 12-lead surface ECG.

## 1. Introduction

Atrial flutter (AFL) is a common type of supraventricular tachycardia (SVT). Based on population studies, it is estimated that there will be annually 200,000 new AFL cases in the US alone [1]. AFL arrhythmia is characterized by electrical signals that regularly propagate along various conduction pathways within the myocardial tissue with self-sustaining mechanisms [2]. Recurrent sustained AFL can lead to significant symptoms such as palpitations, fatigue, syncope, stroke, and even heart attack.

**Figure 1:**
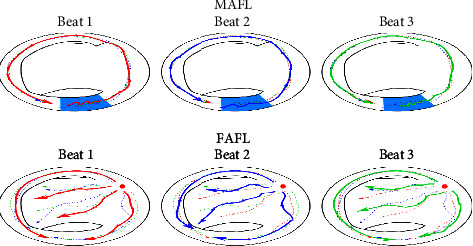
Hypothetical propagation of depolarization wavefront inside the atrium (depicted here: right atrium). The beat number indicates a sequence of atrial beats. Note the path similarity in MAFL, and the lack of it in FAFL.

Atrial flutter can be classified into two different mechanisms, that is, focal AFL (FAFL) and macroreentry AFL (MAFL) according to the characteristics of the conduction propagation [3]. FAFL starts from a single spot, while MAFL is a process that circles a significant obstacle (see Figure 1 for an illustration).

An effective invasive treatment for AFL is radiofrequency catheter ablation (RFCA). It aims to create a conduction barrier to block the reentry of the loop or to destroy ectopic pacemakers. In addition, the list of abbreviations is given in Table 1. During the procedure, intracardiac electrocardiograms, pacing maneuvers, and isochrone mapping are used to characterize the AFL mechanism during RFCA treatment [2, 4, 5]. This can only be performed once the catheters are introduced into the heart.

Although RFCA is preferred for AFL treatment due to its efficacy, its significant dependence on the electrophysiology study makes it a time-consuming and laborious treatment. Therefore, its efficacy can be improved before going under any invasive procedure if the characteristics of AFL (e.g., its mechanism) can be identified from some noninvasive techniques. The noninvasive 12-lead surface ECG is mostly used in clinical detection to differentiate the AFL from atrial fibrillation or normal ECG [6]. The noninvasive differentiation of the AFL mechanisms through 12-lead ECG would help clarify the ablation strategy and reduce the required time and resources in invasive cardiac mapping. However, it has been pointed out that differentiating AFL mechanisms from surface ECG can be a difficult task [7].

Previous attempts are highly dependent on delineation [8] and morphology [3, 9, 10] of atrial waves to discriminate the AFL mechanism through 12-lead surface ECG. Recently, the recurrence quantification analysis (RQA)-based model has been used to distinguish AFL mechanisms from a synthetically generated ECG dataset based on a computational model [11, 12]. Moreover, the authors highlighted the differences in ECG features of FAFL and MAFL.

Classifiers are tailored to differentiate two or many classes using advanced data processing techniques. Their hyperparameters are typically tuned by minimizing some cost function based on a set of given data. However, it most frequently disregards the issue of class prevalence. When the classes are imbalanced (one class more prevalent than others), the classifier's decisions favor the majority class [13, 14]. This affects the classifier's performance, and hence the resulting diagnosis. In medical science, datasets are often imbalanced due to, e.g., rarity of the disease, difficulty in obtaining data, or time and money constraints.

In this study, we propose a classifier model for discriminating the AFL mechanism using peak-to-peak successive atrial activities of 12-lead surface ECG through linear classifiers. Furthermore, the feature extraction of the proposed model is independent of the delineation and morphology of atrial activities.

Due to the class imbalance present in our working dataset, several data augmentation techniques have been used to counteract this. The synthetic minority oversampling technique (SMOTE) [15] is used widely in different fields that create synthetic samples from minority classes to balance the dataset and minimize bias, e.g., article [16]. However, SMOTE has been shown to shrink the variance of the original dataset and introduce correlation between samples [17]. In this study, we also propose a theoretical modification to the original algorithm in order to correct the shrunken variance issue.

The contribution made by the authors in this article is highlighted as follows: (i) methodological improvements to correct the variance shrinkage in classical SMOTE, (ii) a method to parametrize the generation of synthetic P-P intervals in terms of real data, (iii) a novel classifier to differentiate focal from macroreentrant AFL, and (iv) selection of several relevant features that discriminate FAFL from MAFL.

The organization of this article is outlined here.  Section 2 describes the preprocessing of raw data after collection from the hospital and further elaborates on the hypothesis idea. Different standard data augmentation techniques and data generation protocols are explained as feature extraction and selection methodologies.  Section 3 reports the results of data augmentation technique validation to identify the best augmentation rate.  Section 4 discusses the results obtained in the section on the classification results before and after augmentation and the selection of relevant features.  Section 5 concludes the article.

## 2. Materials and Methods

### 2.1. Data Acquisition and Preprocessing

The 61 patients who took part in this study were all under consideration for AFL ablation between January and December 2017 and were registered in a French hospital in Monaco, known as the Centre Hospitalier Princesse Grace. The patients' demography with collection parameters is summarized in Table 2.

All ECGs were obtained during the ablation process using the acquisition system (Boston Scientific, USA). The ECGs were obtained using nine electrodes that were placed on the surface of the body. In contrast, the sampling frequency of the system was set to 2 kHz. The quantization level, which served as the analog to digital conversion resolution, was set to 16 bits. A supine angle was kept the same for all the patients on the surgical table.

In electrophysiological investigations conducted during the ablation procedure, mapping maneuvers establish the target for the ablated source, which distinguishes between the AFL mechanism and other mechanisms, enabling the accurate determination of the ablation spot. The report is made after the ablation. It provides information about the patient's condition, the AFL, the circuit, the orientation, and the direction of the circuit, among other things, which have been used as the ground truth in this study.

**Figure 2:**
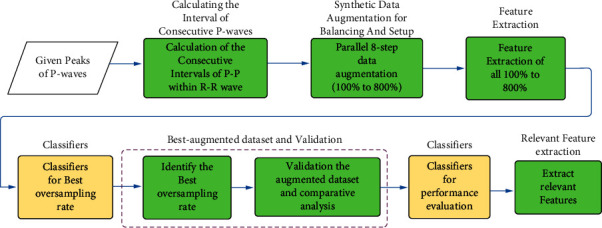
Block diagrams of the proposed methodology. In contrast, the novel contribution of this research is highlighted in green shades.

Consecutive P-waves are required in this study. The ratio between atria and ventricle conduction must be sufficiently large in order to identify these waves, hence we do not include ECGs whose conduction ratio is less than or equal to 2 : 1. Therefore, a limitation ratio is required between atria and ventricles to avoid nonconsecutive issues. With all these limitations, 5 FAFL and 41 MAFL valid records were obtained. These ECGs were then bandpass filtered (passband = [3 : 40] Hz) using two-stage high-pass and low-pass filters with a Chebyshev type II structure. A notch filter at 50 Hz is also used to remove powerline interference. The overall methodology of the proposed research structure is arranged in Figure 2, which can be clearly understood as the novel method used for discrimination of the AFL mechanism.

### 2.2. Calculating the Intervals of Consecutive P-Waves

Atrial flutter can be characterized by electrical signals that repeatedly propagate along various physiological pathways [2, 18]. Macroreentrant and focal AFL have very different activation patterns in the endocardium. One is in the form of a stable reentrant loop, and the other is a point source from which depolarization originates and propagates throughout the entire atrial structure. As shown in Figure 1, the depolarization wavefront patterns in the two mechanisms are quite different. On one hand, it is expected that in MAFL, the stable circuit produces a stable ECG pattern without much variation. On the other hand, centrifugal depolarization in FAFL cannot guarantee that similar paths will be encountered by the wavefront. This instability will translate into varying ECG patterns.

It is hypothesized that the mechanism of AFL can be differentiated from 12-lead surface ECG based on the cycle length variability of the visible consecutive atrial activities (i.e., two or more than two P-waves within R-R interval). Notably, the intervals between P-wave peaks are hypothesized to be more variable in FAFL than in MAFL.

**Figure 3:**
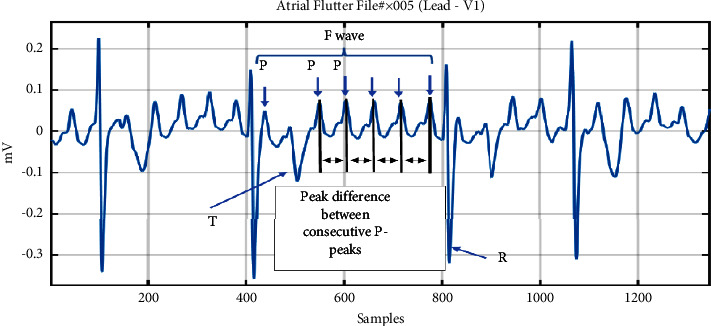
Measures of peak-to-peak intervals between consecutive P-waves within R-R waves in AFL with 7 : 1 ratio.

For each record, the lead containing the largest R-wave energy is selected, and the peaks of atrial activities (P-waves) have been identified by the GLRT method [19]. As shown in Figure 3, intervals between each P-wave have been measured and collected as a series. P-waves overlapped with T-waves have also been considered in this study and are estimated using the least square polynomial estimation [20]. Next, intervals above 300 ms are removed from the series, to ensure that false intervals not related to consecutive P-waves are removed. In total, 444 and 2546 intervals were obtained from 5 focal and 41 macroreentrant AFL ECGs, respectively.

### 2.3. Synthetic Data Augmentation for Balancing

The current dataset presents a heavily imbalanced class ratio of about 8 : 1 for MAFL against FAFL. One can expect the results to be biased towards MAFL (the majority class). Various kinds of data augmentation techniques have been proposed to overcome the imbalance issues present [21]. This study proposes a comparative study among three such techniques to conclude on which one is the best for the considered scenario. These techniques are SMOTE [15, 22], modified-SMOTE, and smoothed-bootstrap. The input to be augmented is the series of P-P intervals.

#### 2.3.1. SMOTE

The main idea of SMOTE is to generate new synthetic interval data based on the linear combination of two interval data *X*_*j*^th^Interval_ and *X*_*j*^th^Interval_^*k*^ where the latter is one of the *k*-nearest neighbors of the former. The synthetic data is then an interpolation within the sample space in a defined neighbourhood. The new synthetic interval is defined as

(1) $$\begin{array}{c} S_{J^{th}Interval}=X_{J^{th}Interval}+\alpha \left(X_{J^{th}Interval}^{k}-X_{J^{th}Interval}\right)\text{,} \end{array}$$

where *S*_*J*^th^Interval_ is the synthetic interval and *α* is a random number belonging to [0, 1]. The result is a synthetic interval data that is randomly generated along the line between *X*_*J*^th^Interval_ and *X*_*J*^th^Interval_^*k*^. In our scenario, we consider the neighborhood to encompass the totality of the dataset instead of a local neighborhood (i.e., *k* = *N* − 1 assuming there are *N* examples in the dataset).

#### 2.3.2. Modified-SMOTE

Some theoretical properties of SMOTE for high-dimensional in-class imbalanced data have been discussed in [17]. One such property is that the synthetic samples have the same mean as the original dataset, but its variance is shrunk by a factor of 2/3. To counteract the shrunken variance, we propose the following modification to (1):

(2) $$\begin{array}{c} S_{J^{th}Interval}^{Modified}=X_{J^{th}Interval}+\frac{3}{2}\alpha \left(X_{J^{th}Interval}^{k}-X_{J^{th}Interval}\right)\text{,} \end{array}$$

where all the terms are as defined previously. The additional coefficient 3/2 reexpands the variance of the newly augmented data to match the original dataset. The complete derivation and concept have been derived in Appendix (A.1). The pseudocode for the algorithm of modified-SMOTE is shown in Algorithm 1.

#### 2.3.3. Smoothed-Bootstrap

The bootstrap is a conventional method based on resampling with replacement from a conveniently small dataset to construct bootstrap datasets. These derived datasets serve to estimate different functionals of the original distribution. However, the bootstrap distributions are typically discrete. For example, the cumulative distribution function of the classic bootstrap is

(3) $$\begin{array}{c} F_{Bootstrap}\left(X\right)=\overset{N_{B}}{\underset{j=1}{\sum }}\frac{\theta \left(X-X_{j}\right)}{N_{B}}\text{,} \end{array}$$

where *θ* is the Heaviside step function or unit step function, *X* is the distribution value of the minority class, and *N*_*B*_ is the size of the bootstrap dataset. Smoothed-bootstrap allows to smoothen the distribution and renders it continuous. First, the original points are randomly shifted before every resampling [23]. A specially chosen continuous kernel function *f*_*j*_ is used as follows:

(4) $$\begin{array}{c} F_{Smoothed}\left(X\right)=\overset{N_{B}}{\underset{j=1}{\sum }}\frac{f_{j}\left(X-X_{j}\right)}{N_{B}}\text{,} \end{array}$$

and thus it renders the distribution continuous. Data points can then be sampled from this distribution. In this research, a nonparametric kernel density function is used.

### 2.4. Data Augmentation Setup

**Figure 4:**
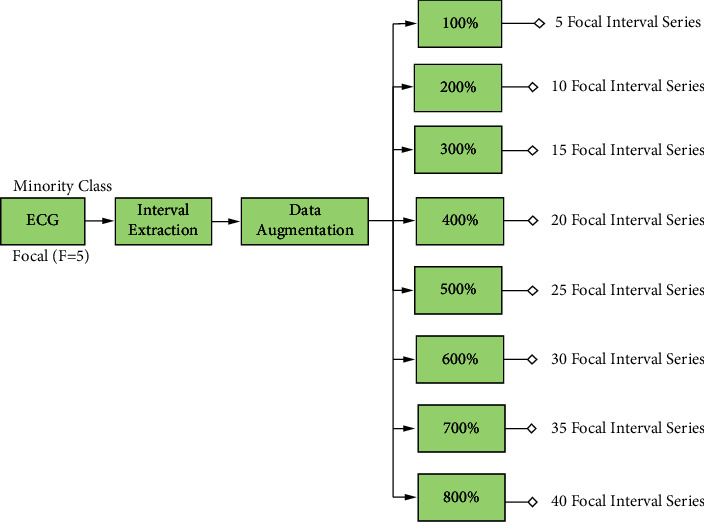
Flow of synthetic data generation.

The minority class (FAFL) is used in Figure 4 to generate synthetic data. The interval series of each ECG record were used. For each original interval series, an interval is randomly selected and used to generate a synthetic interval, based on the three algorithms. The process is repeated until the number of synthetic intervals matches that of the current original interval series. This is then repeated for all FAFL ECG records until the desired augmentation rate is achieved (e.g., 200% augmentation rate means each of the five FAFL ECG record generates two synthetic interval series, for a total of 10 synthetic interval series).

It is known that misuse of data augmentation can lead to biased performance as synthetic samples introduce nonnatural qualities (e.g., correlation). It is then a question about how much synthetic data one should introduce. To analyze the effect of augmentation on classifier performance as well as on the best augmentation rate, this study considers rates from 100% (twice the size of the original dataset) until 800% (9 × the size of the original dataset) in steps of 100%. Note that at an 800% oversample rate, the minority class becomes exactly balanced with the majority class.

### 2.5. Feature Extraction

The characteristic features describing the interval series have been divided into the following four categories concerning the data: central tendency, dispersion, shape, and length. A total of 10 features were considered and is summarized in Table 3. Due to the difference in mechanism, it is expected that FAFL and MAFL would display different values for these features.

### 2.6. Feature Selection Method and Classifiers

Feature selection aims to avoid issues due to large complexity classifier models (e.g., overfitting and cost ineffectiveness) and improve its performance by selecting only relevant features. We consider in this study two approaches to feature selection, that is, (1) the filter method, using Wilcoxon's rank-sum test to determine the significant difference in data medians, and (2) the wrapper method, by evaluating all possible feature combinations (1023 combinations) and determining, for each feature, the feature score. The score is determined as follows:

For every combination length (e.g., single feature and pair of features), a score of 1 is assigned to a feature if it was found to participate in the combination with the maximum accuracy, for that particular combination length. The scores for all combination lengths are added and normalized by the number of features available. Relevant features are those whose scores are closest to 1, and vice versa. The algorithm of both the filter method and the wrapper method is shown in the form of pseudocode in Algorithm 2. The wrapper method allows the evaluation of the relevancy on combinations of features and accounts for possible interactions amongst them, in contrast to the filter, which can only evaluate features one by one.

Three linear classification models: Linear Discriminant Analysis (LDA), Logistic Regression (LOG), and Support Vector Machine (SVM) have been used. Note that nonlinear classifiers have not been considered to avoid further issues related to overfitting due to the scarcity of data.

## 3. Results

### 3.1. Validation of the Data Augmentation

**Figure 5:**
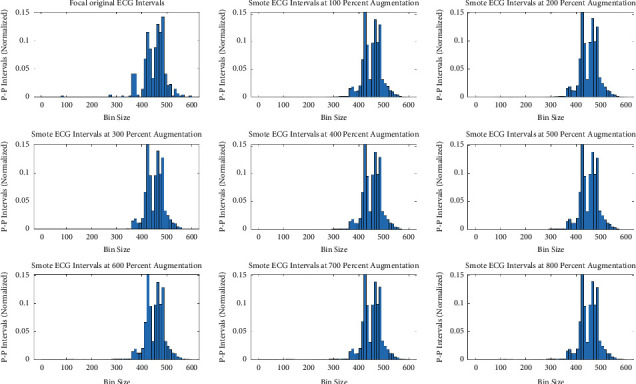
Augmented minority (focal) ECG by SMOTE.

**Figure 6:**
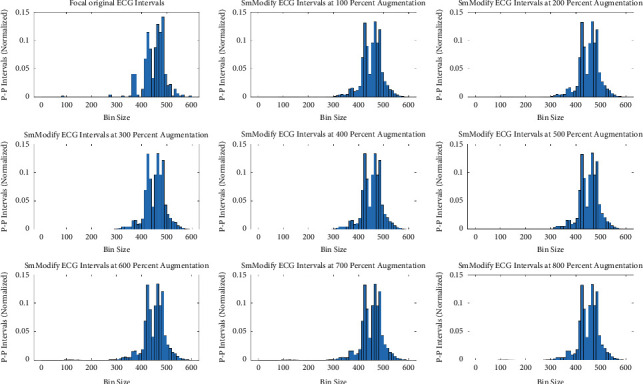
Augmented minority (focal) ECG by modified-SMOTE synthetic technique.

**Figure 7:**
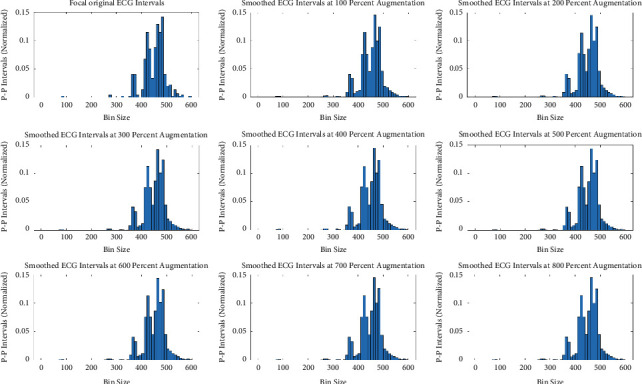
Augmented minority (focal) ECG by smoothed-bootstrap synthetic technique.

The original minority class distribution has been used to generate synthetic and augmented distribution using three different data augmentation techniques. The comparison has been simplified into the following four tests for validation. For each of these tests, 100 augmented datasets were generated for each augmentation rate (i.e., 100% to 800%), and the test results were averaged. Unless otherwise stated, the augmentation rate is fixed to the best rate, which is 400%. However, the impact of data augmentation from the minority class of five focal ECGs (in terms of intervals) in eight equal steps is demonstrated in Appendix B Figures 5–7.

#### 3.1.1. Graphical Exploratory Analysis (CDF and Boxplot)

**Figure 8:**
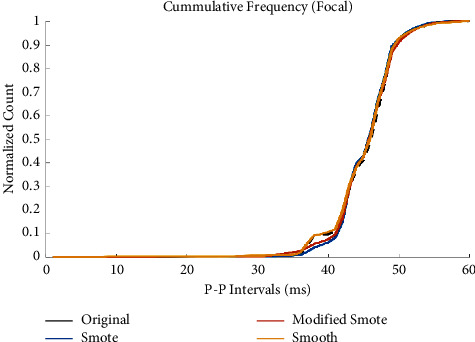
Empirical CDF of the original and augmented P-P intervals of all FAFL records at best augmentation rate (400%).

The average empirical cumulative distribution function (CDF) is shown in Figure 8 for all three augmentation techniques. The original empirical CDF is shown for comparison. The bin size was set to 5 ms arbitrarily. All three techniques correctly follow the original pattern. Smoothed-bootstrap follows the original CDF most closely. Modified-SMOTE and SMOTE present some skewness in the range from 180 ms to 205 ms. However, modified-SMOTE presents less skew compared to SMOTE.

**Figure 9:**
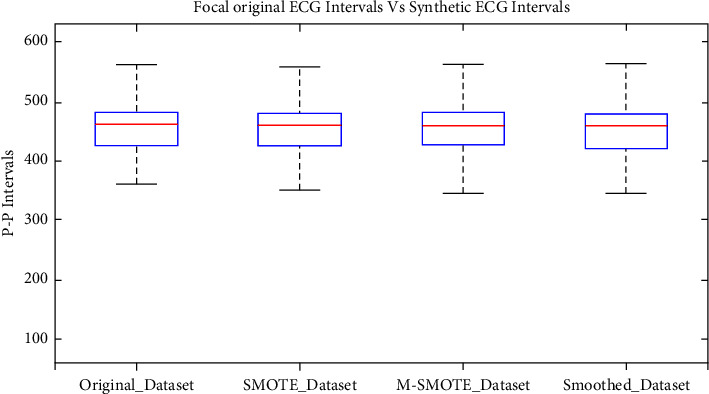
Comparison of 400 percent augmented focal.

Figure 9 shows the average box plot for all three techniques along with its original focal intervals. It has been observed that the central quartile (50%, median) of all three augmentation techniques has the same value as each other and is relatively close to the original dataset. As a quantitative comparison, the median difference in quartile values between the original and each of the three augmented datasets is calculated and summarized in Table 4. The difference in all augmented dataset medians with the original is very small. However, modified-SMOTE has been found to match the original regarding upper and lower quartile ranges (75% and 25%). This shows that modified-SMOTE is a better technique.

#### 3.1.2. Nongraphical Exploratory Analysis (Descriptive Statistics and the Goodness-of-Fit Test)

**Figure 10:**
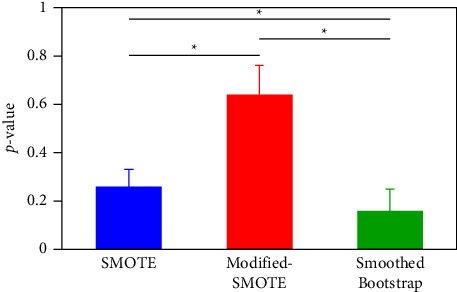
Comparison of Kolmogorov F02D Smirnov test p values at the best augmentation rate. Asterisks indicate significant differences between augmented datasets (*t*-test, *p*p < 0.05).

The Kolmogorov F02D Smirnov goodness-of-fit test has been used to measure the degree of disagreement between the empirical CDFs of the original and augmented datasets. The p value of this test was taken as a measure of similarity (higher values theoretically mean higher similarity).  Figure 10 summarizes the statistics of the p values. Modified-SMOTE had the largest p value (0.64 ± 0.12), suggesting a very high distribution similarity to the original. SMOTE and smoothed-bootstrap have significantly smaller values than this, with smoothed-bootstrap being the smallest (average 0.26 ± 0.07 vs. 0.16 ± 0.09).

Finally, three descriptive statistics, namely, (1) mean, (2) variance, and (3) skewness of the augmented dataset were compared to the original. The difference between original and augmented dataset statistics was calculated and shown in Table 5 as percentages of the original value. The minimum differences are marked in bold font. It can be observed that smoothed-bootstrap has replicated the closest variance and skewness to the original dataset, whereas modified-SMOTE has the minimum difference in the mean only.

### 3.2. Selection of the Best Augmentation Rate

**Figure 11:**
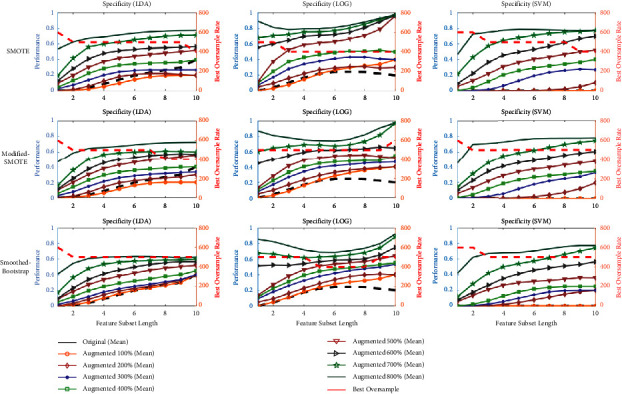
Specificity of proposed augmentation techniques by three classifiers (LDA, LOG, and SVM) for all feature subset lengths. Right *y*-axis indicates performance of the classifier using synthetic samples, and left *y*-axis shows the best oversample rate for a particular feature-length (red line).

Figure 11 shows the maximum performance in terms of specificity of the three classifiers, for each feature combination length. The ideal rate would be the one that maximizes accuracy, sensitivity, and specificity (considering MAFL as the target class). However, under difficult conditions such as heavy class imbalance, these measures have to be considered carefully. Since the imbalance here affects the negative class (i.e., FAFL), we propose to trade off better specificity against lower sensitivity.

To identify the best augmentation rate, the average curve of all augmentation rates was calculated for each feature combination length. The augmentation rate, whose curve has the minimum overall distance from the average curve was chosen as the best augmentation rate. The best rate is shown in red dashed lines in Figure 11 (referring to the right axis). It is observed that the best rate is not uniform across the different combination lengths. It is primarily stable between 400% and 600%. We have selected a lower range of 400% as the best augmentation rate to prefer the minimum synthetic ratio compared to 500% and 600%.

### 3.3. Performance Evaluation by Linear Classifiers for Best Oversampling Rate

**Figure 12:**
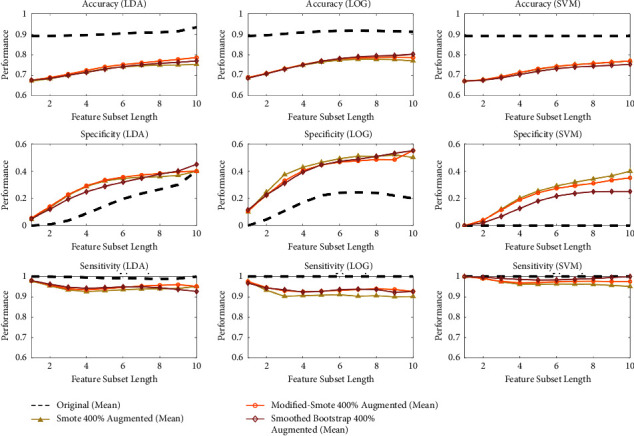
Performance evaluation of the proposed augmentation techniques by three classifiers (LDA, LOG, and SVM) for all feature subset lengths.

Three linear classifiers LDA, LOG, and SVM, and their performances are shown in Figures 11 and 12. One common behavior is that the specificity has increased with an imbalance reduction between FAFL and MAFL. Contrarily, sensitivity, and accuracy decreased with increasing augmentation rate.

The maximum performance at 400% augmentation is summarized in Table 6 and shown in Figure 12. The LOG classifier has the highest performance among the linear classifiers, with mean values of 76.13%, 41.42%, and 93.76% for accuracy, specificity, and sensitivity, respectively, and for all three augmentation techniques. The LDA classifier has the maximum performance under modified-SMOTE, with 73.86%, 29.51%, and 95.20% accuracy, specificity, and sensitivity, respectively. Classifier sensitivity has improved after augmentation. The improvement in performance shows that minimizing the imbalance has a positive impact on performance.

### 3.4. Relevant Feature Selection Methods

Two kinds of supervised feature selection methods have been used for identifying the relevant features that can differentiate MAFL from FAFL. The Wilcoxon rank-sum *p* values (filter method) and the feature scores (wrapper approach) are shown in Tables 7 and 8 for the original and best-augmentation rate, respectively. Relevant (grey highlighted) features can be seen to have high feature scores. Despite the *p* values being nonsignificant for most features in general, it can be seen that the wrapper can highlight features that most probably perform better when combined.

## 4. Discussion

The 12-lead surface ECG remains a staple tool for heart disease diagnosis. However, it is rarely used for AFL mechanism diagnosis. On the other hand, it is widely used to distinguish AFL from atrial fibrillation [6]. The proposed methodology thus represents a contribution in the use of 12-lead ECG as a tool for AFL mechanism discrimination. This in turn allows clinicians to have an early insight into the ablation strategy, thus economizing time and resources.

Three classifiers LDA, LOG, and SVM, have been used to evaluate the performance of the original dataset. The accuracy, specificity, and sensitivity obtained are 91%, 17%, and 100%, respectively, for the logistic regression classifier, with similar results for two other linear classifiers. It has been observed that the specificity in all three classifiers exhibited abysmal performance (*<* 20%). This was caused by the heavy class imbalance where there was a 1 : 8 ratio of focal AFL to macroreentry AFL data record. Classifier bias on the majority class cannot be avoided. Therefore, data augmentation techniques were used to minimize the imbalance. At 400% augmentation, the LOG classifier achieved an accuracy, specificity, and sensitivity of 76%, 40%, and 94%, respectively.

The augmentation here does not serve to “improve” the classifier performance: rather, the focus was to “regularize” the obvious bias due to imbalance. It can be seen that despite a drop in overall accuracy, sensitivity did not drop significantly and yet specificity increased more than twice. This suggests that the use of augmentation helps to estimate the correct performance in regard to classifying focal AFL.

### 4.1. Validation of the Data Augmentation

The results of all four tests of data validation are summarized in Table 9. It is initially difficult to identify the uniformly best technique since all three techniques have competing ranks. However, modified-SMOTE has never been listed as rank 3. Therefore, it can be suggested that modified-SMOTE is overall a better technique for data augmentation among the proposed three techniques.

It can be seen in Table 5 that modified-SMOTE produces datasets with generally less variance compared to SMOTE, as suggested in Appendix A. The issue of variance shrinkage was highlighted by Blagus and Lusa [17], but no solution was proposed. Our original contribution here produces a general tool for generating a new dataset with similar first-order and second-order moments to the original dataset. It can be helpful for other researchers in handling imbalanced datasets, which is a real problem in the biomedical field.

### 4.2. Selection of Relevant Features

The relevant features that differentiate the mechanism of AFL with the highest performance at 400% augmentation have been extracted from two different feature selection methods. The results of the filter method and the wrapper approach are already shown in Table 8. It is challenging to decide the relevant feature with a single method since many feature subsets have scored more significantly than the arbitrary threshold of 0.8. Contrarily, it is simple to decide the relevant features in the filter method as only one is significant. However, this method compares single features and ignores dependencies.

One solution is to compare the two methods to conclude on feature relevance. According to this, three relevant features are highlighted (in order of decreasing relevance): F10: sum of all consecutive intervals, F8: the minimum difference between consecutive P-wave intervals, and F5: variance.

A peak-to-peak interval of two consecutive P-waves contains two temporal information, that is, the P-wave duration, defined as the time length from its onset until its end, and the isoelectric line duration. Hence, there is an influence of P-wave morphology in our measured P-P interval. Therefore, the variation in both P-wave and isoelectric line duration, due to conduction path variability, contributes both to the differentiation of focal and macroreentrant AFL. The sum of all consecutive intervals (F10) has been selected as the relevant feature based on this hypothetical phenomenon, and it has been found to be different for focal AFL from macroreentrant AFL (22.77 ± 12.13 vs. 14.15 ± 10.57, respectively, *p* *<* 0.05). Furthermore, the minimum peak-to-peak interval length (F8) discriminated the AFL mechanism based on fast and slow conduction velocity (393.68 ± 54.51 vs. 424.63 ± 74.48, respectively).

In summary, the sum of all consecutive intervals is the relevant feature to discriminate the mechanism. Finally, the acceptance of our study's hypothesis about the flutter mechanism has also led to the conclusion that the variable feature is a more relevant subset for distinguishing the atrial flutter mechanism.

### 4.3. Performance Evaluation by Linear Classifiers

It has been concluded from the previous section that the best-augmented ratio is 400%, and the modified-SMOTE is the appropriate technique for augmentation. Therefore, the performance of the proposed method has been conducted at 400% of the modified-SMOTE by using five-fold cross-validation. Its results are shown in Table 10 concerning accuracy, specificity, and sensitivity. These results validate that the consecutive intervals of P-P peaks are the significant factors for the discrimination of the AFL mechanism from 12-lead surface ECG.

### 4.4. Comparative Analysis

The definitions of AFLs and a new classification correlated with mechanisms were proposed in 2001 by an international panel of specialists [3]. They have briefly explained the tachycardia mechanism concerning mapping, transient entrainment, and ECG pattern. According to them, during AT, the presence of isoelectric lines indicates the presence of underlying focal mechanisms in a vast majority of patients. In contrast, the lack of isoelectric lines indicates the presence of macroreentry mechanisms in a vast majority of patients during AT. Importantly, it is also possible to observe isoelectric lines in macroreentry, however, only if significant atrial scarring is present. This statement paves the way for researchers in focal and macroreentrant atrial flutter cases. An extensively wide study is focused on isoelectric intervals in discriminating the focal from macroreentrant by invasive and noninvasive procedures. However, limited research was found in noninvasive mechanisms for discrimination of the AFL mechanism, especially the 12-lead surface ECG, discussed here and it is summarized in Table 11.

Two methods for discriminating focal from macroreentrant atrial flutter were proposed by Brown et al. [8]. First, the P-wave duration of the focal should be less than 160 ms (accuracy, 80%), and second, the ratio of P-wave duration to tachycardia cycle length should be less than 45% (accuracy, 95%). This model is highly dependent on the delineation of atrial activities to calculate the cycle length of each P-wave, which requires a high signal processing technique. In contrast, our results were measured from the proposed consecutive P-waves, which were measured from the atrial activity peaks within the R-R intervals. Therefore, the proposed model keeps safe from the advanced signal processing and discriminates the atrial flutter mechanism without requiring the delineation of atrial activities (specificity, 76.88%). Moreover, the original dataset percentage ratio between macroreentrant and focal was 65 : 35, whereas in our study case, it was 89 : 11. After augmentation it became 67 : 33.

Three relevant features were extracted by Chang et al. [10] based on PWM (P-wave morphology): lower voltage in macroreentrant as compared with focal (1.3 ± 0.3 vs. 1.5 ± 0.2 mV, *p*p = 0.02); high incidence of the positive polarity of lead V6 in focal (88% vs. 55%, *p* = 0.03); and longer cycle length in focal (296 ± 107 vs. 224 ± 25 ms, *p*p = 0.01). This case was performed experimentally based on a retrospective analysis and, similarly, required advanced signal processing for morphological analysis of atrial activities. In contrast, the proposed model is directly independent of the delineation and morphology of atrial activities. However, our proposed model includes the cycle length of atrial activity and isoelectric interval without advanced signal processing into the measured consecutive P-P interval within the R-R interval. In detail, the consecutive P-P interval has three pieces of information, such as (i) the approximate second half cycle of the first P-wave, (ii) is the isoelectric interval between consecutive p-waves, and (iii) approximately the first half cycle of the second P-wave. We identified the sum of all consecutive intervals (F10) as a relevant feature extracted from our proposed model, which discriminates between focal and macroreentrant AFL (22.77 ± 12.13 vs. 14.15 ± 10.57), respectively.

Recently, a study generated synthetic datasets through eight torso models using twenty different original AFL mechanisms [11, 12]. They produced 1,256 sets of 12-lead ECG records through a forwarding solution. Furthermore, six RQA-based characteristics were retrieved using two approaches, revealing that a 12-lead surface ECG can characterize the differentiation between FAFL and MAFL. With this in mind, we have generated synthetic ECG intervals from the available minority dataset, which contains non-CTI-based left and right circuits. We generated ECGs in the feature space (consecutive P-P interval) instead of the standard time domain because oversampling techniques used in this model worked in feature space. Our results show that at the best-oversampling rate, the minimum P-P interval length (F8) discriminates the AFL mechanism based on fast and slow conduction velocity (393.68 ± 54.54 vs. 424.63 ± 74.48, respectively).

### 4.5. Limitation and Future Works

This study is based on the variation of intervals between two consecutive atrial activities. At least two atrial activities must be visibly present between the ventricle activity in the ECG. In terms of ratio, it can also be said that the ratio of atrial and ventricle activity must be greater than 2 : 1. This criterion is oftentimes strict and renders the data collection a burdensome task. The selection of a maximum delay between the two P-waves is based on assumptions. It should ideally be set after consultation with several electrophysiologists since slower rates can be observed. Further clinical data should be added to handle the imbalance issue in the study dataset.

In this study, the modification of the SMOTE algorithm for correcting variance shrinkage was performed, assuming that the random multiplier *α* was drawn from a uniform distribution of the modified range. This was proven to theoretically preserve the original moments of the data distribution up to the second moment. It is an open question about what other distributions may be considered to preserve other data properties. This research can also be extended by exploring more valuable classifiers after balancing the dataset with new samples as future work.

The proposed modified-SMOTE was evaluated on two different mechanisms to validate the modification. First, we have theoretically proven the concept of the modification of the classical SMOTE. Then, we performed a comparative analysis of the modified algorithm, classical algorithm, and other oversampling techniques on the real dataset. However, the performance of modified-SMOTE should be analyzed on public datasets to compare and validate its results with other kinds of SMOTE modification.

## 5. Conclusion

This noninvasive study helps identify the AFL mechanism using 12-lead surface ECG, which allows insight into the disease before the catheter ablation procedure. Consecutive intervals of P-waves are hypothesized to contain crucial information regarding the AFL mechanism. Our findings indicate that they are helpful in the discrimination of focal AFL and macroreentry AFL, which does not rely on advanced signal processing such as the measure of the delineation, onset, and offset of the atrial waves. This study has also applied several data augmentation strategies to cure class imbalance in the original dataset. Based on a classical algorithm, a novel augmentation method (modified-SMOTE) was modified to correct a theoretical issue present in the original algorithm.

Three linear classifiers have been used to discriminate against the AFL mechanism. At the best augmentation rate of 400%, the logistic regression classifier achieved an average sensitivity, specificity, and accuracy of 90.24%, 49.5%, and 76.88%, respectively. It was concluded that the sum of all consecutive atrial activities is a relevant feature to differentiate the AFL mechanism.

## Figures and Tables

**Algorithm 1:**
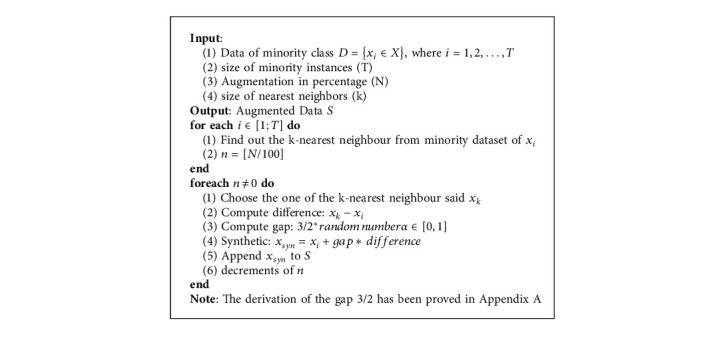
Algorithm of modified-SMOTE.

**Algorithm 2:**
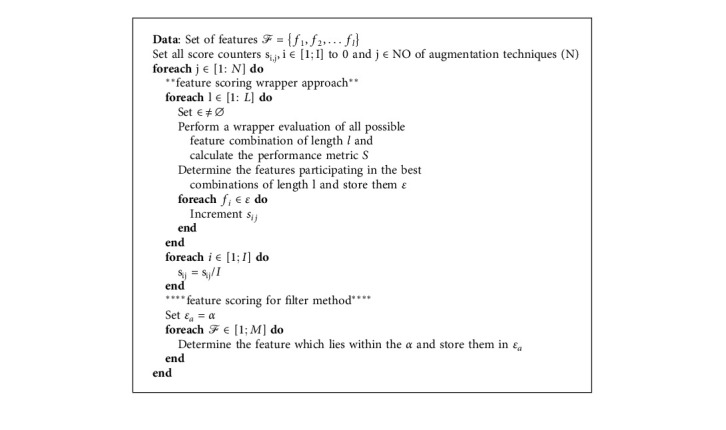
Feature scoring algorithm.

**Table 1:** List of abbreviations.

AFL	Atrial Flutter
CDF	Cumulative distribution function
CL	Cycle length
FAFL	Focal atrial flutter
GLRT	Generalized likelihood ratio test
MAFL	Macroreentrant atrial flutter
LDA	Linear discriminant analysis
LOG	Logistic regression
PWM	P-wave morphology
RFCA	Radiofrequency catheter ablation
RQA	Recurrence quantification analysis
SVM	Support vector machine
SVT	Supraventricular tachycardia

**Table 2:** Patients' demography.

Variable	Focal AFL	Macroreentrant AFL
Cycle length (msec)	230 ± 22.36	248.10 ± 39.38
Before cardiac surgery or ablation	3/5	22/41^*∗*^
Left circuit	2/5	19/41
NonCTI	5/5	19/41^*∗∗*^

(^*∗*^) three ECGs are undefined and (^*∗∗*^) one ECG is defined.

**Table 3:** Feature list.

		Statistics	Description
F1	Central tendency	Mean	Average value of a set
F2		Median	Middle value of a set
F3		Mode	Most common value of a set

F4	Dispersion	Standard deviation	Spread of the values in a set
F5		Variance	

F6	Shape	Skewness	Distribution asymmetry
F7		Kurtosis	Distribution tailedness

F8	Length (interval)	Maximum	Largest value of a set
F9		Minimum	Smallest value of a set
F10		Sum	Sum of all values of a set

**Table 4:** Difference of original dataset from augmented dataset at 400% augmentation rate.

Quartile (%)	SMOTE	Modified-SMOTE	Smoothed-bootstrap
25	0	0	6
50	1.5	1.5	1.5
75	2	0	3

**Table 5:** Comparison in the percentage of the augmented dataset by descriptive statistics on the minority data (focal AFL).

	Mean			Variance			Skewness		
	SMOTE	Modified	Smoothed-bootstrap	SMOTE	Modified	Smoothed-bootstrap	SMOTE	Modified	Smoothed-bootstrap
Difference between statistics of the original dataset and the augmented dataset (in percentage)									
**100**	−0.19	**−0.17**	0.43	25.52	10.65	**−4.36**	18.34	19.98	**3.45**
**200**	−0.19	**−0.14**	0.43	25.74	9.69	**−4.27**	23.87	19.74	**4.69**
**300**	−0.18	**−0.17**	0.42	25.61	10.59	**−4.01**	20.70	18.82	**4.43**
**400**	−0.20	**−0.16**	0.41	25.93	10.59	**−3.83**	20.35	18.92	**4.85**
**500**	−0.19	**−0.17**	0.44	25.95	9.95	**−4.26**	21.51	20.11	**4.51**
**600**	−0.20	**−0.15**	0.41	25.93	10.26	**−3.78**	21.53	20.01	**4.81**
**700**	−0.19	**−0.17**	0.42	25.45	10.77	**−3.97**	21.00	20.14	**4.42**
**800**	−0.19	**−0.17**	0.41	25.85	10.55	**−4.04**	22.86	20.80	**5.15**

There are three augmentation techniques (Smote, Modified-Smote, and Smoothed-Bootstrap) for each parameter (Mean, Variance, and Skewness). The technique with the minimum difference is indicated in bold font.

**Table 6:** Mean value of classifier performance at 400% data augmentation.

Technique	Performance	LDA	LOG	SVM
SMOTE	Accuracy	72.48	75.22	72.76
	Specificity	28.38	41.42	23.30
	Sensitivity	94.29	91.54	96.81

Modified-SMOTE	Accuracy	73.86	75.82	72.89
	Specificity	29.51	39.70	21.41
	Sensitivity	95.20	93.76	97.85

Smoothed-bootstrap	Accuracy	72.96	76.13	71.80
	Specificity	27.94	40.36	15.93
	Sensitivity	94.83	93.56	99.13

**Table 7:** Feature selection methods (both filter and wrapper) at the original dataset.

	Original dataset			
	Filter method	Wrapper method		
Features	p value	Feature score		
		LDA	LOG	SVM
Mean	0.80	0.9	1	1
Median	1	1	1	1
Mode	0.97	0.9	1	1
Std	0.57	0.9	1	1
Var	0.57	0.9	1	1
Skew	0.34	0.9	0.9	1
Kurt	0.62	0.8	0.7	1
PDmin	0.39	1	1	1
PDmax	0.69	0.7	0.8	1
Interval	0.28	0.8	0.9	1

*Note.* In the filter method, we used the Wilcoxon rank-sum test.

**Table 8:** Median and standard deviation values of both filter and feature selection methods on all synthetic techniques at 400% data augmentation.

Median of p value by filter method				Median of feature scores by wrapper method								
Features	SMOTE	Modified-SMOTE	Smoothed-bootstrap	SMOTE			Modified-SMOTE			Smoothed-bootstrap		
				LDA	LOG	SVM	LDA	LOG	SVM	LDA	LOG	SVM
Mean	0.54	0.55	0.55	0.7	0.7	0.8	0.6	0.7	0.7	0.5	0.6	0.8
Median	0.88	0.91	0.84	0.7	0.7	0.8	0.5	0.7	0.7	0.6	0.6	0.7
Mode	0.95	0.94	0.87	0.5	0.7	0.7	0.4	0.7	0.7	0.5	0.7	0.6
Std	0.86	0.40	0.34	0.7	0.6	0.8	0.7	0.7	0.8	0.7	0.7	0.9
Var	0.86	0.40	0.34	0.7	0.6	**0.8**	**0.8**	0.7	**0.8**	0.7	0.65	**0.8**
Skew	0.11	0.11	0.28	0.6	0.7	0.8	0.6	0.7	0.7	0.6	0.7	0.7
Kurt	0.51	0.74	0.76	0.7	0.8	0.9	0.7	0.3	0.8	0.7	0.6	0.7
PDmin	**0.22**	**0.15**	**0.13**	0.7	0.7	**0.9**	**0.8**	**0.9**	**0.9**	**0.8**	**0.9**	**1**
PDmax	0.27	0.58	0.46	0.5	0.6	0.7	0.6	0.6	0.7	0.5	0.6	0.7
Interval	**0.01**	**0.01**	**0.01**	**1**	**1**	**1**	**1**	**1**	**1**	**0.9**	**1**	**1**
Standard deviation of p value by filter method				Standard deviation of feature scores by wrapper method								
Mean	0.055	0.068	0.109	0.114	0.132	0.082	0.113	0.095	0.116	0.115	0.155	0.071
Median	0.051	0.071	0.095	0.110	0.088	0.089	0.107	0.096	0.090	0.090	0.111	0.096
Mode	0.049	0.068	0.091	0.137	0.090	0.110	0.117	0.123	0.138	0.114	0.130	0.188
Std	0.078	0.085	0.146	0.089	0.122	0.116	0.122	0.130	0.116	0.106	0.083	0.099
Var	0.078	0.085	0.146	0.077	0.149	**0.084**	**0.064**	0.133	**0.085**	0.083	0.116	**0.072**
Skew	0.071	0.094	0.148	0.125	0.124	0.177	0.100	0.082	0.160	0.130	0.097	0.145
Kurt	0.188	0.180	0.188	0.123	0.176	0.102	0.122	0.141	0.089	0.127	0.112	0.118
PDmin	**0.061**	**0.038**	**0.056**	0.112	0.189	**0.169**	**0.115**	**0.034**	**0.145**	**0.139**	**0.078**	**0.074**
PDmax	0.053	0.089	0.074	0.167	0.098	0.095	0.105	0.115	0.093	0.146	0.126	0.134
Interval	**0.001**	**0.00**	**0.002**	**0.010**	**0.031**	**0.010**	**0.026**	**0.038**	**0.024**	**0.050**	**0.014**	**0.080**

The relevant features have been emphasized, and the use of bold font indicates that certain sets of features have achieved greater significance than the arbitrary threshold of 0.8.

**Table 9:** Summary of data validation results at best augmentation rate.

Parameter	Subparameter	Rank-1	Rank-2	Rank-3
CDF		Smoothed-bootstrap	Modified-SMOTE	SMOTE
Boxplot		Modified-SMOTE	SMOTE	Smoothed-bootstrap
KS test		Modified-SMOTE	SMOTE	Smoothed-bootstrap
Statistical	Mean	Modified-SMOTE	SMOTE	Smoothed-bootstrap
	Var	Smoothed-bootstrap	Modified-SMOTE	SMOTE
	Skew	Smoothed-bootstrap	Modified-SMOTE	SMOTE

**Table 10:** Performance evaluation by linear classifiers at 400% data augmentation with five-fold cross-validation.

Technique	Performance	LDA	LOG	SVM
Modified-SMOTE	Accuracy	77.81	76.88	77.45
	Specificity	41.35	49.50	36.25
	Sensitivity	95.60	90.24	97.56

**Table 11:** Related works on discrimination of AFL mechanism (PWM: P-wave morphology, CL: cycle length, and NA: not applicable).

Author	Year	Ratio macro/focal (ECGs)	Technique	Classifier	Parameter (significant)	Performance
Brown et al.	2007	27/14	PWM, CL	NA (autocorrelation performed)	PP < 160 ms P/CL < 45%	P: Sensitivity: 90% Specificity: 90% P/CL: Sensitivity: 86% Specificity: 98%

Chang et al.	2011	51/17	PWM CL	NA (empirical-based study)	V6 > 0.9 mV, CL > 265 ms V6 < 0.9 mV, CL > 290 ms	Accuracy focal: 93% Accuracy macro: 88% Accuracy focal: 100% Accuracy macro: 100%

Luongo et al.	2020	Original: 11/9 Augmented: 1256	Recurrance quantification	Decision tree (DT), KNN, and radial basis neural network (rbNN)	RQA-based features	Hit rate: 67.7%

Proposed model		Original: 41/5 Augmented: 15 (focal)	Consecutive P-P intervals (within R-R interval)	LDA, LOG, and SVM	Sum of all consecutive intervals (*p*p < 0.05)	Accuracy: 76.88% Specificity: 49.50 Sensitivity: 90.24%

## Data Availability

The data used to support the findings of the study can be obtained from the corresponding author upon request.

## Appendix

### A. Proof of Synthetic Sample Variance Shrinkage in SMOTE and a Countermeasure

Let *X*_*j*_, *X*_*j*_^*k*^ ∈ *R*^*M*^ be two *k*-near samples from a dataset *χ* of *N* samples. According to SMOTE [15], a new synthetic sample *S*_*j*_ is generated according to the following rule:

(A.1) $$\begin{array}{c} S_{j}=X_{j}+\alpha \left(X_{j}^{k}-X_{j}\right)\text{,} \end{array}$$

with *α* as a random number from the standard uniform distribution.

The expression in (A.1) can be rearranged as follows:

(A.2) $$\begin{array}{c} S_{j}=X_{j}+\alpha \left(X_{j}^{k}-X_{j}\right)\\ =X_{j}+\alpha X_{j}-\alpha X_{j}^{k}\\ =\left(1-\alpha \right)X_{j}+\alpha X_{j}^{k}\text{,} \end{array}$$

which makes it clear that the synthetic sample is an interpolation between *X*_*j*_ and *X*_*j*_^*k*^. In light of the bootstrap sampling theory [24], *α* can be understood as a “smoothing” factor, allowing the selection of new samples that are not directly within *χ*. A crucial part of this procedure is to preserve the properties of the probability distribution, such that new samples *S* have the same first-order (mean) and second-order central (variance) moments.

In what follows, we consider each *X* as a realization of a random variable *ξ* distributed according to some probability distribution. We then consider the following equation:

(A.3) $$\begin{array}{c} \sigma_{j}=\left(1-A\right)\xi_{j}+\xi_{j}^{k}\text{,} \end{array}$$

with *ξ*_*j*_*, ξ*_*j*_^*k*^ i.i.d., and *A*∼*U* (0, 1). Note that this is related to (A.1) when we assume *X*_*j*_, *X*_*j*_^*k*^, and *α* are the realization of the respective random numbers, then *S*_*j*_ can thus be considered an application of the values of the previous. The implication from this consideration is that all *x* ∈ *χ* are distributed according to a common probability distribution. In addition, *ξ*_*j*_^*k*^ could be any one neighbor of *ξ*_*j*_. Hence, in such a setup, we consider all samples to be nearest neighbors (i.e., *k* = *N* − 1).

The form in (A.3) resembles a mixture distribution with uniformly distributed mixing weights. Assuming that *ξ*_*j*_, *ξ*_*j*_^*k*^, and *A* are all independent, we have *E*[*σ*_*j*_] = *E*[*ξ*_*j*_] and Var[*σ*_*j*_]=(2/3)Var[*ξ*_*j*_]

Proof We resort to the law of total expectation which is

(A.4) $$\begin{array}{c} \begin{array}{c} E\left[\sigma_{j}\right]=E_{A}\left[E_{\xi }\left[\left(1-A\right)\xi_{j}+A\xi_{j}^{k}\left|\alpha \right.\right]\right]\\ =E_{A}\left[E_{\xi }\left[\left(1-A\right)\xi_{j}+A\xi_{j}^{k}\right]\right]\\ {=E}_{A}\left[\left(1-A\right)E_{\xi }\left[\xi_{j}\right]+AE_{\xi }\left[\xi_{j}^{k}\right]\right] \end{array}\\ =E_{A}\left[\left(1-A\right)E_{\xi }\left[\xi_{j}\right]+E_{A}\left[A\right]E_{\xi }\left[\xi_{j}^{k}\right]\right]\\ =E_{A}\left[1\right]{-E}_{A}\left[A\right]E_{\xi }\left[\xi \right]{+E}_{A}\left[A\right]E_{\xi }\left[\xi \right]=E\left[\xi \right]\text{.} \end{array}$$

Then, we resort to the law of total variance, that is,

(A.5) $$\begin{array}{c} \begin{array}{c} Var\left[\sigma_{j}\right]=Var\left[E_{k}\left[\sigma_{j}\left|\alpha \right.\right]\right]\\ =Var\left[E\left[\xi \right]\right]+E_{A}\left[V\right.ar\left[\left(1-A\right)\right.\xi_{j}+A\xi_{j}^{k}\\ =0+E_{A}\left[{\left(1-A\right)}^{2}Var\left[\xi_{j}\right]+A^{2}Var\left[\xi_{j}^{k}\right]\right]\\ =Var\left[\xi \right]E_{A}\left[{\left(1-A\right)}^{2}+A^{2}\right]\\ =Var\left[\xi \right]E_{A}\left[1-2A+2A^{2}\right] \end{array}\\ =Var\left[\xi \right]\left(1-2E_{A}\left[A\right]+2E_{A}\left[A^{2}\right]\right)\\ =\frac{2}{3}Var\left[\xi \right]\text{.} \end{array}$$

It can be seen that the quadratic expression 1 − 2*E*[*A*]+2*E*[*A*^2^] introduces the shrinkage factor in the variance. To correct this, the following problem can be a random number *B*∼*F* such that 1 − 2*E*[*B*] + 2*E*[*B*^2^] = 1. The approach in this study assumes that *F* = *U* (0, *b*) where *b* is to be determined. The expression then evaluates to

(A.6) $$\begin{array}{c} 1-b+\frac{2}{3}b^{2}=1\text{.} \end{array}$$

The roots of the abovementioned equation is *b*={0; (3/2)}. Naturally, we pick the solution *b*=(3/2). In order to implement this change inside the SMOTE algorithm (see [15] for the details), the factor *α* can be replaced with $\tilde{\alpha }=b\alpha$.

### B. Related Figures

The effect of data augmentation from the available original minority of 5 focal ECG intervals in 8 equal steps is shown in Figures 5–7 where 5 ms is taken as the bin size of the histogram.
